# What's in a Name?—Consequences of Naming Non-Human Animals

**DOI:** 10.3390/ani1010116

**Published:** 2011-01-19

**Authors:** Sune Borkfelt

**Affiliations:** Institute of Language, Literature and Culture, Aarhus University, Aarhus, Denmark; E-Mail: engsb@hum.au.dk; Tel.: +45-40-58-64-18

**Keywords:** animal, nonhuman animal, naming, animal names, labels, representations, power relations

## Abstract

**Simple summary:**

History teaches us that the act of naming can have various consequences for that which is named. Thus, applying labels as well as both specific and generic names to non-human animals can have consequences for our relationships to them, as various examples show. The issues of whether and how we should name other animals should therefore be given careful consideration.

**Abstract:**

The act of naming is among the most basic actions of language. Indeed, it is naming something that enables us to communicate about it in specific terms, whether the object named is human or non-human, animate or inanimate. However, naming is not as uncomplicated as we may usually think and names have consequences for the way we think about animals (human and non-human), peoples, species, places, things etc. Through a blend of history, philosophy and representational theory—and using examples from, among other things, the Bible, Martin Luther, colonialism/imperialism and contemporary ways of keeping and regarding non-human animals—this paper attempts to trace the importance of (both specific and generic) naming to our relationships with the non-human. It explores this topic from the naming of the animals in Genesis to the names given and used by scientists, keepers of companion animals, media etc. in our societies today, and asks the question of what the consequences of naming non-human animals are for us, for the beings named and for the power relations between our species and the non-human species and individuals we name.

It is an interesting fact that representations are not just expressions, but also impressions. That, even when we as recipients regard representations as fact or as mere entertainment, they typically come loaded with preset ideas of the world, certain values, subjective perceptions and conceptions, which can then work their way into our own ideas and conceptions, consciously or subconsciously. The narratives of European explorers and ‘discoverers’ during the 15th and 16th centuries illustrate this perfectly. Many of them include what we today know are often grossly exaggerated or even purely fictional accounts of lands, peoples and animals supposedly found on non-European continents (dog-headed cannibals, disproportionately gigantic snakes, elephants, predators and so on) [1,2]. Nevertheless, they were often presented to Europeans at the time as true accounts of explorations and thus ultimately helped form European perceptions and conceptions of the rest of the world, which arguably helped lay the ground for later ‘civilizing’ measures and actions taken [3].

The abovementioned aspect of representations is one of the main reasons why the study of representations—of literature, films, art—is profoundly important and interesting. Representations are not only expressions of our thoughts and attitudes; they are also influences, often in more ways than we tend to notice. This includes, of course, the act of naming, which is arguably the most basic representation of something or someone and should therefore be of interest to anyone, who studies (or indeed anyone who uses) representations of any kind. As European explorers could do the inhabitants of other continents a disservice by representing them in certain ways, so we may do favours or disservice to places and beings (human and non-human) when we name them. A name is a representation and can therefore potentially carry all the values, ideas, perceptions and conceptions carried by representations and have the array of potential consequences, which can ensue from representation.

Naming is, in a way, the very first and most basic act of language, because it is what enables us to talk or write about something in specific terms. If language is, as has been argued, a means of power—providing a “technique for knowing” places, people, animals, and things [4]—then naming is at the very centre of this power. When naming, for instance, an individual animal or a species, we not only choose how we want to represent that animal, but also how others are to represent and perceive it: we lay the foundations of representations and perceptions to come. This makes naming a powerful tool of control. To return to the language of imperialism and colonization, this is a power that is, as Stephen Greenblatt informs us, manifested in a very real way through giving islands and countries the names of kings and queens in Europe, an example being the powerful expression of values in Columbus' naming the first American island he encountered “*San Salvador*, in remembrance of the Divine Majesty, Who marvelously bestowed all this” [5].

In the example of Columbus and San Salvador, not only does his act of naming signify his taking possession of the island on behalf of his God and the Spanish Monarchy, it also signifies that he thinks himself righteous in doing so, despite the fact that it has a native population, who have already named it, as he well knows: “the Indians call it ‘Guanahani’”, he writes, thus cancelling native nomenclature to assert his power [6].

When dealing with the naming or re-naming of newly encountered lands and peoples by Europeans as an act of power over (the perception of) what is named, it seems there is an obvious comparison to be made with the relationship between humans and other animals, since we both name other animals (specifically and generically) and demonstrate our power over them in a number of ways. A power that is often, especially in European cultures, thought of as part of a ‘natural’ or even God-given order of things. Thus, in ‘Genesis’ Adam (*i.e.*, Man), much like Columbus naming the islands he encounters, is given the power to name the animals: “And out of the ground the Lord God formed every beast of the field, and every fowl of the air; and brought them unto Adam to see what he would call them: and whatsoever Adam called every living creature, that was the name thereof. And Adam gave names to all cattle, and to the fowl of the air, and to every beast of the field” [7].

This naming of the animals clearly shows a divide between Man and the other animals. Adam, gifted with the language and understanding to name what he sees, is given the power for his very first act of control over the animals by God: the act of naming and thereby defining the beings named. An act that is second only to creation. As Erica Fudge states, “[i]t is as if the animals had no identity, no presence without Adam, and their inherent powerlessness, perhaps most easily described as their inability to name themselves, has persisted in human relations with animals. An animal cannot think, we argue, and therefore it is down to us to think for it” [8]. Naming, thus, is symbolic of the unequal power relations inherent to our relationship with other species. And indeed, commentators throughout history have most often taken this inequality for granted. Martin Luther, for instance, also comments on the act of naming the animals as something showing a great wisdom naturally inherent to man (but not to the other creatures):

Here again we are reminded of the superior knowledge and wisdom of Adam, who was created in innocence and righteousness. Without any new enlightenment, solely because of the excellence of his nature, he views all the animals and thus arrives at such a knowledge of their nature that he can give each one a suitable name that harmonizes with its nature [6].

If one follows Luther's reasoning, this is indeed an amazing understanding of the nature of animal species, as Adam sees the animals as what they are and names them accordingly. On the other hand, it could also be argued that Adam through the act of naming actually decides the nature of each animal, showing thereby his power over it. Indeed, where the first act after the creation of the animals in the second book of ‘Genesis’ is Adam's naming them, the first thing that comes after their creation in a parallel passage in the first book is the assertion of Man's right to rule over the animals: “and let them have dominion over the fish of the sea, and over the fowl of the air, and over the cattle, and over all the earth, and over every creeping thing that creepeth upon the earth” [9]. Luther also sees the naming of the animals as a symbol of this power:

From this enlightenment there also followed, of course, the rule over all the animals, something which is also pointed out here, since they were named in accordance with Adam's will. Therefore by one single word he was able to compel lions, bears, boars, tigers, and whatever else there is among the most outstanding animals to carry out whatever suited their nature [6].

To name the animals is thus an assertion of rule over them, an act whereby Man makes the animals, their actions and their use, subjects to his power, appointed to him by God. A process very similar indeed to Columbus' naming of islands God has enabled him to discover, and indicative of the same power relations justified by reference to divine will. It deserves mention as well, though, that some theologians prefer to think of a gentler relationship between humans and animals and therefore interpret the act of naming in a different way. F. B. Welbourn, for instance, has written that “[b]y the act of naming the animals man recognized his responsibility towards them” [10]. But of course this still presupposes an inherent powerlessness on behalf of the animals and therefore a natural inequality between humans and animals. Naming still signifies inequality and dominance, it is merely questioned what humans should do with this God-given power.

Naming is thus not only the first and most basic of linguistic processes; it is also an excellent example of the power or control that is in many ways inherent to language use. Whether what is named are lands, people or animals, the process of naming reflects the worldview of the one who names rather than the view of what is named. Thus, when Europeans have wanted to describe or name something in parts of the world outside Europe, this has most often been done through some form of reference to something already found on the European subcontinent, even in cases where the likeness implied by the new names do not seem altogether straightforward. This applies to lands and people, but is perhaps most obvious in the descriptions and names of animals.

Both the anthropocentrism and eurocentrism of animal names like ‘Thomson's gazelle’ or ‘Lichtenstein's hartebeest’ is quite obvious, but a certain eurocentrism is in fact inherent to many other names of non-European animals as well, due to the references to animals already known in Europe, which are included in the names. The name ‘hippopotamus’, for example, derives from the Greek *hippos* (horse) and *potamós* (river), yet there is little resemblance between the hippopotamus and the horse its name compares it to, or rather the resemblance that may have been perceived by whoever named the animal is a product of that person's (European) imagination and background. The word entered the English language as late as the sixteenth century, perhaps without much contemplation of its etymology [11,12].

Another example is the ‘guinea pig’, which as it happens has little to do with neither Guinea nor pigs, yet is in many European languages likened to pigs in one way or another [13]. Such a comparison to an animal found in the ‘Old World’ to which the guinea pig actually bears little resemblance or kinship is obviously Eurocentric, but it is also anthropocentric as it names the animal by a human reference to another animal. Moreover, if the ‘guinea’ in guinea pig is merely symbolic of something distant or exotic, as seems likely given that the animal is not found in Guinea, this further contributes to the anthropocentric othering of the little animal as well as to the Eurocentric idea of what is, and what is not, distant or exotic. Today, a metaphorical use in English of the expression ‘guinea pig’ for an object of experimentation furthermore makes it an interesting subject for discussions on linguistic anthropocentrism [16].

Of course far from all animals in the ‘New World’ or other colonial parts of the world were named or described by reference to known species of the ‘Old World’. Some were described in ways that served what José Rabasa calls “a European inclination to represent and classify American fauna within the monstrous” and/or were the subjects of descriptions that played a part in the “formalization of particular features first derived from oral or written sources”, which is of course no less Eurocentric [17]. Finally, some names were adapted from what appeared to the Europeans to be the names ascribed to them by the native humans of the colonized lands. Yet in this adaptation, the written language of the Europeans was used to Latinize the native expressions and teach the natives how the names should be written. Moreover, the transcription of such native expressions into European languages must surely in some cases, if not in most, have caused a shift in pronunciation as well. Native names for animals, as well as for other things, which have been adapted in this way, have thus played a role (some may say still play a role) in a kind of cultural imperialism exerted over native humans in colonial and postcolonial societies.

In Genesis, Adam is of course naming species of animals rather than individuals as, coincidentally, did European explorers and scientists who ‘discovered’ new animal species on other continents. Such naming, of course, leaves little room for variations between distinct animal societies, not to mention individual variations between single animals. Thus, for us as humans, the vast majority of other animals seem to have fit nicely into generic categories, and our use of these categories may say quite a lot about the ways in which we regard other species.

Bundling together a number of individuals in order to create hierarchies and assert power relations is a practice that has of course been applied to humans time and again throughout history, whether through reference to race and ethnicity, gender or other shared features of those being categorized and put at the lower end of a hierarchy. Through all ages, those who have physically resembled us the most have been those most willingly admitted into our communities and those, who we have wished to keep beyond the moral pale have been categorized generically by reference to the features perceived to mark their difference from us.

With categorizations of non-human animals, however, we might argue the practice has been taken to its extreme. Indeed, using the very term ‘animal’ to bundle together all other species is perhaps exactly the most extreme example of generic naming in terms of how many differences there are between the creatures defined by it. And the exclusion of ourselves from that same generic term, by viewing the word ‘human’ as an opposite to it, is as arbitrary as would be the exclusion of any other species. Moreover, as Tim Ingold has argued, all the qualities we as humans are claimed to “uniquely have, the animal is consequently supposed to lack; thus, the generic concept of ‘animal’ is negatively constituted by the sum of those deficiencies” [18]. As a consequence, being an ‘animal’ becomes intrinsically negative and helps to keep those defined as such outside the moral community.

Other categorizations work in similar ways. We regard, and treat, animals differently, depending on the category they belong to. Thus, for instance, what is thought of as the proper way of treating a rabbit may differ remarkably depending on whether we have labelled it as a ‘pet’, as ‘vermin’, as a ‘food animal’ or as a ‘research subject’. Thus, labelling and categorizing can give us the power of applying non-humans (often from the same species) to different and often contrastive uses without taking the arbitrariness of such a practice into account. And of course there are a number of other labels or categorizations, which we apply to (groups of) animals and which tell us how we are to regard individual animals or species that come under such a label [19].

Indeed, if we wish to be able to regard an animal or species in a certain way, one could argue we allow ourselves to do so by labelling it in a way that fits our intended use. For instance, it was convenient for European hunters in early twentieth century Kenya, that the Kenyan Game Department—a governmental institution set up to regulate hunting—sometimes listed even lions as ‘vermin’ while “baboons, zebra, bush pig and hyena were regularly listed on the vermin schedule and could be shot on sight” [22].

Given the various ways we use other animals according to the labels applied to them, as discussed above, it seems safe to say that—except perhaps in the case of a few categories, which we regard favourably—our use of generic names and labels have done other species few favours. In addition, of course, such use generally disregards the potential individuality of non-human animals, unless a certain label—‘pet’, for instance—suggests to us that an animal belongs to a category where individual names are in order. Non-human animals, according to tradition, are irrational and soulless and are therefore not individuals in the sense that humans are.

Nevertheless, we do name other animals individually in many situations, and the question we may ask ourselves is if the unequal power relations implied by generic naming also come into effect when naming individuals. In addition, we might ask what other consequences being named may have for the individual non-human animal.

As Vicki Hearne argued in her 1986 book *Adam's Task*, typography has given us ways of distancing ourselves from other animals:

Typography has also made possible further gaps between us and animals, because we have become able to give them labels without ever calling them by name. The registered names of most horses and dogs are primary examples. Champion Redheath Gunner, C.D., C.D.X., U.D., for example is not a name but something halfway between labels (of the sort found on packing lists or in livestock inventories) and titles … like the titles of books. Such names are bookkeeping [23].

In addition, she argued, we can know the difference between a name with ‘scare quotes’ around it, “Annie”, and one without such typographic markers, Annie [24]. In the first case, the quotation marks imply to us that the name is not a real name the way our own, human, names are. It is used *about* an individual animal, but is not part of that animal's individual identity in the way that a human name is. Similarly, according to Hearne, if we merely use a name to speak about an animal but not directly to it, we are not allowing the animal to have an identity the way we have and are therefore upholding an artificial distance between ourselves and the animal. And since, she argued, some animals are able to know their own name, thus having a consciousness of their own identity, we may do them a disservice if we do not name them. For Hearne, the tool to overcome this distance is animal training:

Obedience-training horses creates a logic that demands not only the use of a call name, since the imperatives demand it, especially for the command ‘Dobbin, Come!’, but also the removal of the quotes from the name, the making of the name into a real name rather than a label for a piece of property, which is what most racehorses' names are [25].

Thus, Hearne pointed out an essential problem in choosing not to name other animals—that we may use it to uphold the idea that they are different from us in a way that is somehow essential to how we think about the world. In fact, Hearne seems to imply almost a duty to train (and thus name) those animals who are susceptible to it, calling “obedience training” a “sacred and poetic rather than a philosophical or scientific discipline” [26]. Ultimately, though, the training seems to benefit the human rather than the animal: if an animal knows its name, then it is easier to train and command for the *human* trainer. Thus, even for someone like Hearne, giving names to other animals is still connected to our power over them.

As Hearne pointed out, giving an animal a name does often draw it closer to us. It can make us think about it as an individual, a person. In a way, the animal may become humanized. Especially since we often give non-human animals, who we regard as special in some way, names we would also use for humans. Indeed, it can be argued that as we have, historically, come to regard other animals with greater complexity (and consider, for instance, that they might be able to think or feel), it has become more common to use human names for them. Thus, Keith Thomas has argued that non-human animals have historically been given human names only when they were somehow special and that the tendency to give pets human names became far more common in England during the eighteenth century than it had been before [27].

Today, of course, such practice is quite common. Giving a companion animal or an animal in a zoo, for instance, a human name is a way of showing that an animal is somehow especially dear to us or of acknowledging its closeness to us. But curiously, this approximation does not seem to go both ways, at least in Western or Judeo-Christian cultures. We are still generally unwilling to give human children ‘animal’ names. We will call a dog ‘Jamie’ but tend to shrink away from (or not even consider) calling a human child ‘Rover’, ‘Sparkles’ or ‘Snowflake’, for example. That is, on one hand we are generally willing to ‘elevate’ non-humans especially dear to us to a ‘human’ or ‘near-human’ level through naming, but on the other we hesitate, or seem to uphold a certain taboo against it, when it comes to ‘lowering’ ourselves to an ‘animal’ level in the same way [28]. Surely, this tells us something interesting about our relationships with other animals.

For the individual animals we choose to name, even if they are not conscious about it, being named can mean the difference between life and death. For instance, when a live calf was discovered below a pile of slaughtered cattle during the 2001 outbreak of the foot-and-mouth disease in Great Britain, and was named Phoenix, there was a public outcry on its behalf and it eventually managed to escape the culling policy applied to thousands of other healthy animals during the same outbreak. As Erica Fudge observes, the “anonymity of the slaughter of cattle was disrupted by a calf with a name. Suddenly, what was absent—the individual—became overwhelmingly and powerfully present” [29]. Similarly, the polar bear cub Knut, born in 2006 at the Berlin Zoo, received massive media attention and became famous after being ‘adopted’ by a human caretaker and named. In this way he may have escaped the label of ‘surplus animal’ applied to many other animal cubs born in zoos and later killed when there is no room, or need, for them [30]. In both cases the name is instrumental to people recognizing the animal as an individual and regarding him or her with special fondness and this can influence the fate of an animal significantly. Of course, this is not necessarily always a positive thing. For example, as in the case of Knut, the name may also entail commercialization and a life deeply abnormal for the species of animal in question. Thus, claims have been made that the huge crowds outside his enclosure while he was growing up may have damaged Knut mentally, one activist and zoologist even suggesting that he had turned into “a psychopath addicted to human attention” [31].

Yet the fact remains: we name individual animals when we regard them as special and it is often an expression of fondness, which may end up being subversive to practices that can be seen as harmful to the animal. We regard an animal with a name differently and sometimes an individual animal with a name—even a fictitious animal—may function as an ambassador and change perceptions of an entire species, as one can argue Flipper may have done for dolphins or the 1995 film *Babe* did for pigs. Indeed, in the latter case, the film has been used to argue that people should become vegetarian, whereby one could argue its named protagonist becomes an ambassador for different treatment of a wide range of animal species [32].

Perhaps for the same reason, people who make a living from using, and potentially harming, non-human animals, are often quite critical of naming them and speak of the dangers of anthropomorphising animals. Thus, when the chief manager of the Copenhagen Zoo, Bengt Holst, was asked if the names of the zoo's chimpanzees were not humanising the animals, he admitted:

Det er rigtigt, at et navn er med til at menneskeliggøre et dyr. Og med chimpanserne er det en tradition, som er svær at komme til livs. Det erkender jeg blankt. Egentlig burde vi give dem et nummer eller en benævnelse … [i] respekt for dem, men også for at fortælle den rigtige og sande historie til publikum [33].

[It is true that a name humanizes an animal. And with our chimps it is a tradition, which is difficult to get rid of. I frankly admit that. Actually, we ought to give them numbers or labels … out of respect for them, but also in order to tell our audience the true and accurate story.]

For Holst, it seems, the ‘accurate’ and ‘true’ story about the animals is that they are not like us, are not persons, and do not have the kind of identity, which could warrant a name; a story that arguably makes it easier to justify keeping them in cages. Similarly, as Arnold Arluke tells us, the ‘codes’ used to refer to animals used for experiments “are labels rather than names. This distinction is important: labels are classifications of inanimate objects, as opposed to words used to designate living beings” [34]. Thus, as Vicki Hearne stated with regard to trained animals, actively choosing not to name can be used to distance ourselves from animals and thereby hide or ignore their individuality. Indeed, Arluke reports that persons working with laboratory experiments on animals “reported that they prefer using a number to a name because it gives them more distance from the animal” [34]. We ‘give’ ourselves a distance, arguably ‘taking away’ that same approximation from the animal, in order to justify harming or killing him or her.

Ultimately, then, naming seems to leave us with some dilemmas, depending on the relationship we wish to have with other animals. If we wish to argue, for instance, that non-human animals are worthy of a moral consideration they are generally not afforded today and that their lives as individual animals hold intrinsic value—that they should be subjects rather than objects subjected to our power over them—then the question of whether or not to name them is not altogether clear.

For while naming can be said to be a necessity for language and communication, the very act of naming actually makes animals into objects, which we choose how to perceive, represent and categorize through the names we apply to them. Thus, names can function as a conveyor of certain ideas we have about non-human animals, certain uses we see for them, and thus help determine our behaviour towards them and make them into the objects of certain uses or attitudes, such as ridicule or relative indifference. When we name, we are thus in fact exercising a power over the animals we name, even if we may fundamentally believe our power over them should not determine their lives. On the other hand, not naming can mean distancing ourselves from other animals and disregarding their likeness to ourselves, which makes it easier to justify harmful treatment through reference to the difference between ‘them’ and ‘us’. Which may especially be the case if we choose to apply a label or categorization instead of a name and classify animals as, for instance, ‘vermin’ or ‘research animal’. Indeed, not just denying individual names, but also ignoring the names of species, can help hide a practice, which is harmful to animals: words such as ‘bacon’ and ‘ham’, for instance, may help us ignore the fact that it is in fact an animal, a ‘pig’, we are eating.

Thus, thinking about the nature of names and the way we apply them to other animals may help us better understand our relationships with them. Indeed, it seems, it is imperative that we consider the issue thoroughly if we truly endeavour to understand the ways we perceive, represent and, often as a consequence, ultimately act toward non-human animals.
